# Épidémiologie et facteurs de risque de la prématurité dans le nord-ouest algérien

**DOI:** 10.11604/pamj.2024.47.183.40657

**Published:** 2024-04-12

**Authors:** Nabila Heroual, Nacera Boukfoussa, Leila Houti

**Affiliations:** 1 Centre Hospitalo-universitaire d´Oran, Oran, Algérie,; 2 Labsis, Faculté de médecine Université Oran 1, Labsis, Algérie

**Keywords:** Naissances prématurées, facteurs de risque, santé périnatale, Preterm births, risk factors, perinatal health

## Abstract

**Introduction:**

les naissances prématurées continuent d´être la principale cause de mortalité infanto-juvénile, mais aussi des handicaps sensorimoteurs et des difficultés neurodéveloppementales dans le monde. Le taux d´accouchement prématuré est en augmentation notamment en Algérie. L´objectif de ce travail est de déterminer la fréquence des naissances prématurées dans la wilaya d´Oran et d´identifier les facteurs de risque.

**Méthodes:**

nous avons exploité les données d´une étude transversale multicentrique réalisée dans l´ensemble des 13 maternités publiques de la wilaya d´Oran incluant les parturientes ayant accouché d´un enfant né vivant et/ou mort-né dont l´âge gestationnel était supérieur ou égal à 24-36 semaines d´aménorrhées révolues et le poids ≥500 grammes. Les facteurs démographiques, médicaux et socio-comportementaux de la mère ont été relevés. Une régression logistique a été élaborée pour étudier les facteurs prédictifs de prématurité.

**Résultats:**

le taux d´accouchement prématuré était de 9,9% (45/452). L´âge moyen des parturientes était de 30,4 ± 6 ans. Les grossesses multiples représentaient 2,2% des naissances. Les facteurs liés à la prématurité étaient la menace d´accouchement prématuré (ORa=4,68; IC95%: 2,27-9,64), l´absence de suivi médical de grossesse (ORa=2,83; IC95%: 1,83-6,05) et l´hypertension artérielle gravidique (ORa = 3,69, IC à 95%: 1,83-8,8).

**Conclusion:**

le taux de naissance prématurée retrouvé rejoint les taux observés dans les pays voisins. L´étude a permis d´identifier des facteurs prédictifs dont certains sont déjà ciblés par le programme national de périnatalité. Cependant, il reste indispensable de poursuivre les efforts déployés pour améliorer le suivi et la prise en charge des grossesses et des naissances prématurées à tous les niveaux de soins.

## Abstract (en)

**Introduction:**

preterm births continue to be the main cause of infant and child mortality as well as sensory-motor disabilities and neurodevelopmental difficulties worldwide. The rate of preterm births has been rising, in particular in Algeria. The purpose of this study is to determine the frequency of preterm births in the Oran Wilaya and to identify risk factors.

**Methods:**

we used data from a multicentre cross-sectional study carried out in all Public Maternity Hospitals in the Oran Wilaya (13). The study included parturient women who had given birth to a live and/or stillborn child (with birthweights ≥500 g), whose gestational age was greater than or equal to 24-36 weeks of amenorrhoea. Mothers´ demographic, medical and socio-behavioural factors were recorded. Logistic regression was used to study predictors of prematurity.

**Results:**

preterm birth rate was 9.9% (45/452). The average age of patients was 30.4±6 years; multiple pregnancies accounted for 2.2% of births. Factors related to prematurity were the risk of premature labour (aOR=4.68; 95% CI: 2.27-9.64), the lack of clinical monitoring of pregnancy (OR=2.83; CI 95%: 1.83-6.05) and gestational hypertension (aOR = 3.69, 95% CI: 1.83-8.8).

**Conclusion:**

the rate of preterm births is in line with the rate observed in neighbouring countries. The study identified predictive factors, some of which are already targeted by the national perinatal program. However, it is essential to continue to lead efforts to improve the monitoring and management of pregnancies and premature births at all levels of care.

## Introduction

L´Organisation mondiale de la Santé (OMS) définit la prématurité par la survenue d´une naissance avant la trente-septième semaine d´aménorrhée (SA) révolue [1]. On distingue habituellement la prématurité tardive (naissances entre 34 et 36 SA révolues), la prématurité modérée (32 à 33 SA), la grande prématurité (28 à 31 SA) et la très grande prématurité (< 28 SA) [2]. Chaque année dans le monde, quelque 15 millions de bébés naissent prématurément et près d'un million d´enfants décèdent en raison des complications liées à la prématurité [3]. Selon le rapport de l´OMS, les naissances prématurées représentent 11,1% des naissances vivantes, dont 60% ont lieu en Asie du Sud et en Afrique subsaharienne [4]. La répartition géographique des taux de prématurité révèle des disparités entre les pays [5]. Les estimations du taux de prématurité à l´échelle mondiale se heurtent à de nombreuses difficultés, en particulier l´absence de déclaration systématique des naissances, des estimations de terme imprécises et des erreurs de classement entre naissances sans vie et naissances vivantes [6]. Les complications de la prématurité constituent la principale cause de décès pendant la période néonatale [7]. Actuellement, elles représentent la deuxième principale cause de décès après la pneumonie chez les enfants de moins de 5 ans [3,8].

D´origine hétérogène et idiopathique dans 70%, certaines études suggèrent que les facteurs environnementaux jouent un rôle important et que l'interaction génétique-facteurs environnementaux est plausible [9]. Cependant, un grand nombre de facteurs de risque ont été déterminés. Parmi eux, on retrouve les facteurs sociodémographiques maternels tels que le jeune âge de la mère (moins de 18 ans), la petite taille de la mère (<150 cm), les facteurs socio-économiques (faible niveau d´éducation et de revenu), les facteurs gynécologiques et obstétricaux, à savoir les antécédents de prématurité et de fausses couches tardives, la vaginose bactérienne, les grossesses trop rapprochées (<18 mois), les grossesses multi-fœtales et l´assistance médicale à la procréation [10]. Les pathologies chroniques telles que l´hypertension artérielle gravidique, le diabète gestationnel et la consommation de tabac pendant la grossesse sont aussi impliquées [11-13]. En Algérie, des programmes nationaux de lutte contre la mortalité maternelle et périnatale mis en œuvre depuis 1984, ont conduit à une amélioration des indicateurs de mortalité [14]. Cependant, ils restent insuffisants pour atteindre les objectifs du développement durables (objectif 03) [15]. En 2018, les taux nationaux de natalité, de mortalité infantile et de mortalité néonatale étaient respectivement de 24,39‰ (plus de 1,038,000 naissances vivantes), 21,0‰ et 17,1‰ [16]. À Oran, le taux de mortalité infantile dépassait le taux national (26,8‰) [17] et la prématurité reste une cause importante de mortalité néonatale [18]. Ne disposant pas de données nationales, ce travail a pour objectif de déterminer la fréquence des naissances prématurées dans la wilaya d´Oran et d´identifier ses facteurs de risque ainsi que les facteurs prédictifs dans le but de produire des indicateurs sur la santé périnatale.

## Méthodes

**Cadre de l´étude**: la wilaya d´Oran, ville portuaire de la Méditerranée, est située au nord-ouest de l'Algérie. En 2019, la population était estimée à plus de 2 012 727 habitants pour une superficie de 2,114 km^2^. Le secteur public comprend 5 établissements hospitaliers « mère-enfant », un centre hospitalo-universitaire, un établissement hospitalo-universitaire et plusieurs maternités urbaines. Les taux de natalité globale et de fécondité étaient respectivement de 21,1‰ et 81,7‰.

**Type d´étude, critères d´inclusion**: les données recueillies sont issues d´une étude transversale multicentrique menée dans l´ensemble des 13 maternités publiques de la wilaya d´Oran pendant la semaine du 04 au 10 février 2019. Elle a concerné l´ensemble des enfants nés vivants ou mort-nés, si la naissance a eu lieu entre au moins 24 semaines d´aménorrhée (SA). Ont été inclus l´ensemble des enfants nés vivants ou mort-nés, si la naissance a eu lieu entre au moins 24-36 SA révolues et si le nouveau-né pesait au moins 500 grammes à la naissance. Les questionnaires dont l´âge gestationnel n´était pas renseigné ont été exclus.

**Taille de l´échantillon**: la taille de l´échantillon a été estimée sur la base de la fréquence des naissances prématurées dans la wilaya de Sétif (ville de l´Est d´Algérie) qui était de 03,9% en 2012 [19]. Nous avons déterminé une taille minimum d´échantillon en appliquant la formule:


$$n=\frac{Z^{2}P\left(1-P\right)}{e^{2}}$$

avec Z =1,96 pour Þ=5%, p=03,9% et e degré de précision souhaité (fixé à 2%). Ainsi, la taille minimum de l´échantillon était de 360 naissances.

**Collecte des données**: le questionnaire établi a été testé lors d´une pré-enquête réalisée auprès d´une trentaine de parturientes un mois avant le début de l´étude. L´événement observé étant la naissance, un questionnaire était rempli pour chaque enfant en cas de naissances multiples. Ce dernier comprenait deux parties: 1) les caractéristiques sociodémographiques, le suivi médical et le comportement de la femme au cours de la grossesse recueillis lors d´un entretien en face-à-face avec les femmes avant leur sortie de la maternité, 2) les données relatives au déroulement de l´accouchement et l´état de santé de l´enfant à la naissance collectées à partir du dossier médical. Le recueil des données a été réalisé par les sages-femmes préalablement formées. Les facteurs étudiés étaient la parité, les antécédents obstétricaux pathologiques (les antécédents de mort-né, mort in utéro, accouchement prématuré, hypotrophie et grossesse arrêtée) ; les pathologies chroniques (hypertension artérielle, diabète) et infectieuses, surveillance de la grossesse (consultations obstétricales, échographies) et les mesures anthropométriques (poids, taille) avant l´accouchement. Les facteurs sociaux et comportementaux comprenaient le statut professionnel de la mère, le niveau d´études et le tabagisme actif. Un consentement oral a été obtenu auprès de chaque femme ayant accepté de participer à l´étude et les données ont été anonymisées lors de l´analyse. L´approbation éthique a été accordée par la Direction de la Santé de la wilaya d´Oran.

**Traitement et analyse des données**: l'analyse statistique des données a été réalisée sur le logiciel IBM SPSS Statistics 19.0. Les variables quantitatives ont été exprimées en moyenne et écart-types, et les variables qualitatives en effectifs et pourcentages. La comparaison des moyennes a été faite par le test-t de Student et celle des proportions par le test de Khi^2^ (ou celui de Fischer lorsque recommandé). Une régression logistique simple puis multiple a été réalisée pour étudier les facteurs associés à la prématurité. Le seuil de 20% a été choisi pour la sélection des variables incluses dans la régression logistique. Le seuil de signification statistique a été fixé à 5%.

## Résultats

Sur les 462 naissances recensées durant la période d´étude, 98% étaient des naissances vivantes. Les grossesses monofœtales représentaient 97,8% des cas. Le taux d´accouchement prématuré était de 9,9% (45/452). Environ 57,8% des naissances avant 37 SA relevaient de la prématurité tardive et 20,0% de la grande prématurité (28-31 SA ± 6 J). Aucune naissance d´extrême prématurité n´a été relevée. Parmi les naissances vivantes, la fréquence de la prématurité est passée de 08,6% pour l´ensemble de la population à 07,7% pour les naissances uniques. Les jumeaux présentent un taux élevé de prématurité (30%), soit un risque multiplié par 4 (Tableau 1). Quatorze décès (03,0%) ont été enregistrés dont dix naissances prématurées (71,4%).

**Tableau 1:** taux de prématurité selon le type de naissance, Oran 2019

	Total	Prématuré	Fréquence	IC 95%**
**Grossesses**	**452**	45	10,0	7,52-13,06
**Unique**	442	42	09,5	
**Gémellaire**	10	03	30,0	
**Naissances**	**462**	48	10,4	7,92-13,51
**Naissances vivantes**	**451**	39	08,6	6,39-11,6
**Uniques**	**431**	33	07,7	6,39-11,6
Multiples*	**20**	06	30,0	
**Décès**	**14**	10	71,42	
**Morts nés**	11	09	81,8	
**Décès néonataux précoces**	03	01	33,3	

* grossesses gémellaires ; ** IC: intervalle de confiance à 95%

**Caractéristiques sociodémographiques et antécédents obstétricaux des parturientes**: l´âge moyen des parturientes était de 30,4 ± 6 ans. La part des femmes ayant un niveau d´études supérieur était de 23,7% et 17,7% avaient un emploi. Aucune différence significative n´a été observée pour les caractéristiques sociodémographiques ainsi que les antécédents obstétricaux et médicaux, à l´exception d´antécédents de mort-nés (p<0,01) (Tableau 2).

**Tableau 2:** caractéristiques sociodémographiques et antécédents médicaux de la population étudiée, Oran 2019, analyse univariée

Variables, n (%)	Population globale (N=452)		
	Accouchements prématurés n= 45 (10,0)	Accouchements à terme n = 407 (90,0)	P-value*
**Age (ans)**			0,7
**<**18	1 (25,0)	3 (75,0)	
19-34	33 (09 ,9)	30 (90,1)	
≥35	11 (09,7)	102(90,3)	
**Niveau d'éducation**			0,5
Aucun, Primaire	12 (12,0)	88 (88,0)	
Moyen, secondaire	21 (08,6)	224 (91,4)	
Université	12 (11,2)	95(88,8)	
**Travail pendant la grossesse**			0,09
Oui	12 (15,0)	68 (85,0)	
Non	33 (08,9)	339(91,1)	
**IMC**			0,6
**<**18,5	1 (02,2)	8(01,9)	
19-24,99	17 (37,8)	141(34,6)	
25-29,99	15 (33,3)	189(46,5)	
≥ 30	12 (26,7)	69(17,0)	
**Taille (cm)**			0,4
≤150	3 (09,8)	19 (86,4)	
> 150	42 (13,6)	388 (90,2)	
**Antécédents obstétricaux**			
Mort-né	4 (36,4)	7 (63,6)	**0,01**
Mort in utéro	4(26,5)	11 (73,3)	**0,05**
Antécédent d´accouchement prématuré	1 (12,5)	7 (87,5)	**0,05**
Faible poids de naissance	7(14,9)	40(85,1)	0,2
Grossesse arrêtée	5 (07,5)	62 (92,5)	0,2
**Antécédents médicaux**			
Hypertension	3(14,3)	18 (85,7)	0,4
Diabète	3 (18,8)	13 (81,2)	0,2

* p value: degré de signification (p<0,05)

**Facteurs de risque de la prématurité**: l´analyse univariée des données fait ressortir que l´accouchement prématuré était lié à la menace d´accouchement prématuré (p<10-3), l´hématome rétroplacentaire (p<10-3), et l´absence de suivi de grossesse (p<0,005), le diabète (p<0,01), le diabète gestationnel (p<0,01), l´HTA gravidique (p<0,01) et la parité (p<0,04) (tableau 3). Cependant, aucune association n´a été retrouvée pour le traitement de l´infertilité, les antécédents de grossesse gémellaire, la consommation de tabac et la présence d´un antécédent obstétrical (Tableau 3). Dans l´analyse multivariée, l´accouchement prématuré était associé à la menace d´accouchement prématuré (ORa=4,68 ; IC à 95%: 2,27-9,64), l´absence de suivi médical de grossesse (ORa=2,83; IC à 95%: 1,83-6,05) et l´HTA gravidique (ORa = 3,69, IC à 95%: 1,83-8,8) (Tableau 4).

**Tableau 3:** facteurs de risque des naissances prématurés, Oran 2019

Facteurs de risque	Total (N)	Accouchement prématuré	Accouchement à terme	p
		n(%)	n(%)	
**Parité**				**0,04**
1	43	7(16,3)	36 (83,7)	
2-3	351	28(08,0)	323(92,0)	
4+	58	10(17,2)	48 (82,8)	
**Traitement de l'infidelité**				0,15
Oui	36	06(16,7)	30 (83,7)	
Non	416	39 (09,4)	377 (90,6)	
**Suivi médical de la grossesse**				**0,005**
Oui	389	33 (08,4)	356 (91,6)	
Non	63	12 (10,0)	51 (80,9)	
**Type de grossesse**				**0,01**
Monofœtale	442	42(09,5)	400(90,5)	
Multiples	10	03(30,0)	10 (70,0)	
**Antécédent de grossesse multiple**				0,4
Oui	5	1(20,0)	4 (80,0)	
Non	447	44(09,2)	403 (92,8)	
**HTA gravidique**				**0,01**
Oui	22	6 (27,3)	16(72,7)	
Non	430	39 (09,1)	391 (90,9)	
**Diabète gestationnel**				**0,01**
Oui	16	5(31,2)	1 (68,8)	
Non	436	40 (09,2)	396(90,8)	
**Menace d'accouchement prématuré**				
Oui	59	15 (25,4)	44 (74,6)	**10-3**
Non	393	30 (07,6)	363 (92,4)	
**Hématome rétroplacentaire**	5	4 (80,0)	1 (20,0)	**10-3**
Oui	447	41 (09,2)	406 (90,8)	
Non				
**Un antécédent obstétrical**				**0,05**
Oui	126	18 (14,3)	108(85,7)	
Non	326	27(08,3)	299(91,7)	
**Consommation de tabac pendant la grossesse**				0,06
Oui	16	4(25,0)	12 (75,0)	
Non	436	4(09,4)	395(90,6)	

* p value : degré de signification (p<0,05)

**Tableau 4:** taux de prématurité et rapports de cotes ajustés selon les caractéristiques maternelles, Oran (2019)

Variable avec p-value<5%	ORa*	IC 95%**	P
**Menace d'accouchement prématuré**	4,68	2,27-9,64	10-3
HTA gravidique***	3,69	1,83-8,8	0,003
**Absence de suivi médical de grossesse**	2,78	1,13-5,98	0,007

* ORa: Odds ratio ajusté ; **IC : intervalle de confiance à 95% ; ***HTA : hypertension artérielle

## Discussion

Il s´agit d´une première enquête sur la santé périnatale dans la wilaya d´Oran incluant toutes les naissances pendant une semaine dans les treize maternités publiques. Les résultats observés soulignent l´importance du taux de prématurité (09,9%) à Oran et ont permis d´identifier les facteurs qui y sont associés. La fréquence de la prématurité retrouvée était de 08,6% des naissances vivantes. Ce taux rejoint celui observé dans d´autres pays tels que la Libye, la Tunisie [20], le Bénin [21] et l´Éthiopie [22]. Des taux plus bas ont été observés dans certains pays développés [8,23-25]. En revanche, dans d´autres pays africains, le taux dépassait 13% en 2016 en Guyane [26] et 15% en Afrique de l´Ouest [27]. Néanmoins, la réduction des taux de mortinaissance (en faveur des naissances prématurées) pourrait conduire à une augmentation des naissances prématurées nées vivantes [28]. Malgré les efforts déployés dans la santé néonatale en Algérie, la prématurité demeure une préoccupation et reste un déterminant majeur de la mortalité périnatale et néonatale. Elle est une des principales causes de décès en période néonatale, notamment à Oran [29]. De même qu´elle serait responsable de la moitié des cas de complications neurologiques à long terme et de coûts élevés engendrés par le diagnostic et les soins délivrés aux enfants prématurés [20]. Une étude canadienne a estimé le coût de prise en charge au cours des dix premières années de la vie à 67 467 $ pour les nourrissons prématurés, correspondant à des coûts nationaux totaux de 123,3 millions de dollars pour les nourrissons prématurés [30]. En plus des complications citées, selon une étude menée auprès des adultes nés prématurément, les nourrissons prématurés seraient vulnérables aux maladies cardiovasculaires et rénales chroniques à l'âge adulte [31]. La survie des prématurés dépend du niveau de développement des soins où le taux de survie dès 25 SA dépasse 50% dans les pays développés [8]. À ce titre, des études de cohortes ainsi que des programmes ont été développés dans plusieurs pays pour améliorer les connaissances sur les causes et les conséquences de la prématurité [32,33]. L´OMS a lancé une stratégie mondiale pour la santé des femmes, des enfants et des adolescents « chaque femme, chaque enfant » (2016-2030) [34]. Huit naissances prématurées sur dix étaient relevées entre 32-36 SA (prématurité tardive et modérée). Ceci a été observé à Tlemcen, une wilaya voisine (77,9%) et dans des pays voisins tels que le Maroc (60,8%) et la Tunisie (67,7%) [20]. Cependant, il est plus bas que celui observé en Thaïlande [35] et en France, respectivement 93,4% et 85% [6].

Les grossesses multiples représentaient moins de 3% des grossesses, avec un taux d´accouchement prématuré de 30%, plus bas que celui rapporté par Goldenberg (60%). La surdistension utérine, entrainant des contractions utérines et rupture prématurée des membranes, est le mécanisme responsable du taux accru d´accouchement prématuré spontané [36]. L´âge moyen des parturientes était de 30,4 ans (+ 6), ce qui témoigne du recul de l´âge du mariage dans notre pays qui est en moyenne de 27,1 ans chez les femmes [37]. Nous n´avons pas observé de lien significatif entre l´âge maternel et le risque de prématurité, alors qu´un sur-risque a été rapporté par plusieurs études, notamment chez les jeunes de moins de 18 ans et les femmes âgées de plus 40 ans [10,35]. Parmi les enquêtées, les femmes âgées de moins 20 ans ne représentaient que 02,7% et l´âge moyen des primipares était de 26,6 ans (+ 4,8). Il faut souligner que plus de 73% des parturientes étaient âgées entre 19-34 ans au moment de l´accouchement. Le niveau de scolarité de la mère a souvent été associé au risque de prématurité [10,38], toutefois le niveau d´étude supérieur est considéré comme protecteur, témoignant des progrès réalisés aux développements social et médical de la santé des femmes [39]. Même si aucun lien significatif n´a été observé dans l´étude, la part des parturientes avec un niveau d´étude supérieur ne dépassait pas les 27%. Si l´emploi permet aux femmes de meilleures conditions sociales et d´accès aux soins, parallèlement travailler pendant la grossesse peut se faire dans des conditions parfois pénibles, voire dangereuses. La part des parturientes employées était de 15,6% et nous n´avons pas observé de différence d´accouchement prématuré entre le groupe d´employées et non-employées. Cette différence a été rapportée par une étude portant sur 13 cohortes européennes et plus de 200 000 couples mère-enfant, ou un faible risque d´accouchement prématuré chez les employées (ORa = 0,86 ; CI 95%: 0,81-0,91) a été retrouvé [40].

L´usage de substances psychoactives (alcool, tabac et drogues) pendant la grossesse représente un enjeu de santé publique important en raison des risques pour l´enfant à naître. L´effet du tabac sur la prématurité a fait l´objet de plusieurs études, il est considéré comme un facteur de risque d´accouchement prématuré. Le taux de tabagisme maternel retrouvé était de 03,5%, plus bas que celui rapporté par une étude multicentrique en France (22,1%) [41]. Néanmoins, aucune différence n´a été observée (p<0,07) alors qu´une méta-analyse datant de 2000 a mis en évidence une association significative entre consommation tabagique et prématurité globale (OR 1,27; IC 95%:1,21-1,33 ; 20 études) avec une relation dose-effet [42]. Cependant, même si le tabagisme féminin reste faible en Algérie avec un taux de 0,4% selon l´enquête nationale de 2016, il reste néanmoins fortement sous-déclaré [43]. Parmi les parturientes ayant eu recours au traitement de l´infertilité (8%), 16% avaient accouché avant 37 SA mais aucune différence significative n´a été observée (p= 0,24) entre les groupes. Alors qu´une étude américaine avait montré que les femmes ayant subi un traitement de fertilité avaient un risque de 26% d´accouchement prématuré [44]. D´autre part, l´utilisation accrue de traitements contre l´infertilité se traduit par des taux plus élevés de grossesses multiples, qui sont un facteur de risque global d´accouchement prématuré. Dans notre étude, aucun cas de grossesse gémellaire n´a été observé chez les parturientes traitées pour infertilité. Par rapport aux comorbidités maternelles, la fréquence du diabète gestationnel était de 03,76%, et celle de l´hypertension gravidique 4,9%. Cette dernière est présente dans 5 à 10% des grossesses [45]. Concernant le diabète gestationnel, l´étude Epifane (France, 2012) rapporte un taux plus élevé (08%) [46]. Une différence statistiquement significative pour les deux pathologies a été observée, contrairement à l´étude de Jiang qui a rapporté une différence significative uniquement pour l´hypertension gravidique (p<10-3) [47]. La menace d´accouchement prématuré (MAP), pathologie associant des modifications cervicales à des contractions utérines régulières et douloureuses (survenant 22 et 36 (SA) révolues), peut évoluer en l´absence de traitement vers l´accouchement. Dans notre étude, 13% des parturientes avaient présenté une MAP et le taux d´accouchement prématuré était de 25%. Selon les études, ce taux varie entre 20-67% dans certains pays africains [48].

L´analyse multivariée a identifié trois principaux facteurs associés à l´accouchement prématuré; la MAP, l´absence de suivi médical et l´hypertension gravidique. Malgré la pléthore de facteurs de risque décrits dans la littérature, la majorité des naissances prématurées ne présentent aucun facteur de risque clair. Une méta-analyse de 4,1 millions de naissances uniques menée dans cinq pays à revenu élevé a indiqué que dans 65% de toutes les naissances prématurées ne présentaient aucun des 21 facteurs de risque prédéfinis [28]. L´étude menée est une étude multicentrique qui a inclus les 13 maternités publiques, ce qui a permis d´obtenir un échantillon de 452 parturientes admises pour accouchements. Cependant les maternités privées n´ont pas été incluses dans l´étude, car ne prenant pas en charge les accouchements prématurés, ce qui peut constituer un biais de sélection. Le recueil des données s´est fait par un groupe de sages-femmes préalablement formées, limitant ainsi le biais d´information.

## Conclusion

Cette étude a permis de déterminer la fréquence de l´accouchement prématuré dans le secteur public à Oran et d´identifier certains facteurs de risque tels que la menace d´accouchement prématuré, l´absence de suivi médical de la grossesse et la présence d´une hypertension gravidique. La prématurité est un enjeu important pour les équipes médicales et pour les parents. Mais aussi un indicateur de choix dans l´évaluation des programmes de lutte contre la mortalité infanto-juvénile. Les étiologies de la prématurité sont complexes et multiples. La compréhension des facteurs de risque et le développement de stratégies curatives et préventives, notamment le renforcement des programmes de surveillance des grossesses, la sensibilisation des parturientes et l´éducation des femmes de façon plus générale, permettront d´améliorer la prise en charge de l´accouchement prématuré. Il reste indispensable de poursuivre les efforts déployés pour améliorer le suivi et la prise en charge des grossesses et des naissances prématurées à tous les niveaux de soins.

### Etat des connaissances sur le sujet

- *Les naissances prématurées sont en augmentation dans le monde notamment en Algérie;*
- *La prématurité est une des principales causes de décès en période néonatale;*
- *Plusieurs facteurs de risque ont été décrits dans la littérature*.

### Contribution de notre étude à la connaissance

- *Déterminer la fréquence des naissances prématurées;*
- *Identifier les facteurs de risque auprès des parturientes*.

## Remerciements

Les auteurs tiennent à remercier toutes les sages-femmes ayant participés à l'étude. Les auteurs reconnaissent également le soutien reçu de la direction de la santé.
